# Evaluation of three sample preparation methods for the direct identification of bacteria in positive blood cultures by MALDI-TOF

**DOI:** 10.1186/s13104-016-2366-y

**Published:** 2017-01-18

**Authors:** Hannah Tanner, Jason T. Evans, Savita Gossain, Abid Hussain

**Affiliations:** 1 Public Health England, Public Health Laboratory Birmingham, Heartlands Hospital, Bordesley Green East, Birmingham, B9 5SS UK; 2 Wales Centre for Mycobacteria, Public Health Wales, University Hospital Llandough, Penarth, CF64 2XX UK

**Keywords:** MALDI-TOF, Blood culture, Bacteraemia, Sepsis, Identification, Microbiology, Clinical

## Abstract

**Background:**

Patient mortality is significantly reduced by rapid identification of bacteria from sterile sites. MALDI-TOF can identify bacteria directly from positive blood cultures and multiple sample preparation methods are available. We evaluated three sample preparation methods and two MALDI-TOF score cut-off values. Positive blood culture bottles with organisms present in Gram stains were prospectively analysed by MALDI-TOF. Three lysis reagents (Saponin, SDS, and SepsiTyper lysis bufer) were applied to each positive culture followed by centrifugation, washing and protein extraction steps. Methods were compared using the McNemar test and 16S rDNA sequencing was used to assess discordant results.

**Results:**

In 144 monomicrobial cultures, using ≥2.000 as the cut-off value, species level identifications were obtained from 69/144 (48%) samples using Saponin, 86/144 (60%) using SDS, and 91/144 (63%) using SepsiTyper. The difference between SDS and SepsiTyper was not statistically significant (P = 0.228). Differences between Saponin and the other two reagents were significant (P < 0.01). Using ≥1.700 plus top three results matching as the cut-off value, species level identifications were obtained from 100/144 (69%) samples using Saponin, 103/144 (72%) using SDS, and 106/144 (74%) using SepsiTyper and there was no statistical difference between the methods. No true discordances between culture and direct MALDI-TOF identification were observed in monomicrobial cultures. In 32 polymicrobial cultures, MALDI-TOF identified one organism in 34–75% of samples depending on the method.

**Conclusions:**

This study demonstrates two inexpensive in-house detergent lysis methods are non-inferior to a commercial kit for analysis of positive blood cultures by direct MALDI-TOF in a clinical diagnostic microbiology laboratory.

## Background

Bacteraemia and sepsis have a high mortality rate and it is crucial to start appropriate antimicrobial treatment as promptly as possible [1, 2]. Conventional identification of organisms causing bacteraemia relies on Gram staining of a positive blood culture followed by solid media subculture for 18–24 h and then confirmatory tests to identify the causative organism. Whilst empirical antimicrobial treatment will have been started at the point of blood culture collection, earlier identification of bacteria in blood culture would enable the medical microbiologist to: more specifically target antimicrobial treatment (based on intrinsic and local patterns of antimicrobial susceptibilities of certain genera or species); instigate further relevant investigations; and inform infection prevention and control measures at an earlier stage which could potentially reduce mortality rates.

Matrix-Assisted Laser Desorption/Ionization Time-of-Flight (MALDI-TOF) is an inexpensive method that can identify a broad range of bacterial species directly from positive blood cultures [3]. Numerous methodologies for preparation of positive blood culture samples for MALDI-TOF have been described with a central aim of producing a bacterial pellet that is free from human blood cells, proteins, and culture medium. This can be achieved by detergent lysis of the human cells followed by centrifugation and washing steps of the bacterial pellet using a commercial kit or various other reagents (e.g. saponin and sodium dodecyl sulphate (SDS) solution) [3–7]. Other methods have utilised serum separator tubes [8–10], differential centrifugation or sedimentation [5, 11–14] and ammonium chloride lysis [15]. A subsequent protein extraction step on the pellet is usually required to obtain high yields and high quality results.

In this study we assessed the performance of a commercial kit (Bruker SepsiTyper), two different detergent lysis reagents (5% saponin solution, 10% SDS solution) and two different MALDI-TOF score cut-off values for the identification of bacteria in positive blood culture bottles.

## Methods

### Study setting

The Public Health Laboratory, Birmingham, UK provides a diagnostic microbiology service to the Heart of England NHS Foundation Trust in Birmingham from whom it receives about 27,000 blood culture samples per year. All blood culture bottles are incubated and microbial growth is detected using the BacT/ALERT^®^ system (bioMérieux). Blood culture media used are BacT/ALERT^®^ SA Standard Aerobic, BacT/ALERT^®^ SN Standard Anaerobic and BacT/ALERT^®^ FA Plus (antimicrobial neutralization) media (bioMérieux). No charcoal containing bottles are used. When blood culture bottles flag positive on the system (indicating microbial growth), aliquots of the blood culture medium are used to prepare Gram stains and agar plate subcultures for identification of cultured organisms. The laboratory routinely uses MALDI-TOF (Bruker MALDI Biotyper system) for identification of bacterial isolates from agar plates. Some bacterial species are confirmed with biochemical tests in addition to MALDI-TOF according to ISO 15189:2012 United Kingdom Accreditation Service accredited methods used for routine clinical diagnostics. Routine clinical diagnostic results reported by the laboratory were considered the definitive result with which direct MALDI-TOF results (see below) were compared.

### Study sample collection

On weekdays, over a four week period in December 2013, all blood cultures from patients ≥18 years old which flagged positive on the BacT/ALERT^®^ (bioMérieux) system and had micro-organisms seen in the Gram stain were selected. All selected blood cultures were prepared for direct MALDI-TOF using three different lysis reagents in parallel (as described below) in addition to routine Gram stain and sub-culture methods.

### Direct identification of micro-organisms in positive blood cultures by MALDI-TOF

Two hundred microliters of each of the three lysis reagents (5% Saponin solution, 10% SDS solution, Bruker SepsiTyper Lysis Buffer) were aliquoted into a new screw-capped microcentrifuge tubes and 1 ml of each positive blood culture was added to each tube. All tubes were vortex mixed for 10 s and then incubated at room temperature for 5 min and centrifuged for 1 min at 13,000*g*. Supernatant was removed and each pellet was re-suspended in either 1 ml MALDI grade water for Saponin or SDS or 1 ml SepsiTyper Washing Buffer and centrifuged for 1 min at 13,000*g*. Supernatant was again removed and each pellet re-suspended in 300 μl MALDI grade water and 900 μl 100% Ethanol followed by centrifugation for 2 min at 13,000*g*. The supernatant was then removed and each sample centrifuged again for 2 min at 13,000*g*. Residual ethanol was then removed and the pellets dried for 10 min at room temperature. Pellet size was then estimated. For small or non-visible pellets, 5 µl of 70% formic acid and 5 µl of 100% Acetonitrile was added. For medium-sized pellets, 20 µl of 70% formic acid and 20 µl of 100% Acetonitrile was added. For large pellets, 50 µl of 70% formic acid and 50 µl of 100% Acetonitrile was added. Pellets were re-suspended thoroughly then each sample was centrifuged for 2 min at 13,000*g*. One microliter of each supernatant was then spotted onto a sample spot of a MALDI target plate and allowed to dry. Two spots were prepared for each sample. One microliter of α-Cyano-4-hydroxycinnamic acid (HCCA) matrix solution (Bruker) was then added to each spotted sample and allowed to dry. Genus and species identification was then obtained using the Bruker MALDI Biotyper system.

### MALDI-TOF data analysis

Spectra were automatically captured and analysed by the Bruker MALDI Biotyper system and database (version 2.0, IVD MALDI Biotyper database 4613). No manual acquisition was carried out. Two MALDI spots were analysed for each positive bottle and the highest scoring spot was selected for data analysis. Two different MALDI scoring methods were used: acceptable genus and species identification if a score ≥2.000 was obtained or acceptable genus and species identification if a score ≥1.700 and the top three matches were concordant. Samples giving scores below the cut off values evaluated were recorded as having no identification by direct MALDI-TOF.

## 16S rDNA PCR and capillary electrophoresis

Samples with discordant results between direct MALDI-TOF and the diagnostic laboratory’s reported identification from agar plates were analysed by 16S rDNA PCR and DNA sequence analysis. DNA sequences were queried against NCBI BLAST [16].

### Data analysis

Within the study period, the first positive aerobic and anaerobic blood culture bottle from each patient was selected for data analysis. The McNemar test was used to evaluate if the differences observed between the three different lysis methods were statistically significant.

All MALDI-TOF testing was carried out without investigators seeing the Gram stain or conventional culture and identification results. At the conclusion of the study period, reported results of culture and identification by the clinical diagnostic laboratory for all positive blood cultures were downloaded from the laboratory information system for comparison with the direct MALDI-TOF results obtained.

## Results

During the four week study period, a total of 199 blood culture bottles from 122 patients flagged positive and organisms were seen on the Gram stain. Of the 199 bottles, 10 were antimicrobial neutralization, 99 were standard aerobic and 90 were standard anaerobic media. Species identified from the individual bottles by direct MALDI-TOF are shown in Table 1. The first positive aerobic and/or anaerobic bottle(s) from each patient was included for further study giving 181 blood cultures (including 63 sets of two bottles) from 122 patients.

**Table 1:** Direct MALDI-TOF results from 199 blood culture bottles by lysis method and cut off value used

Species identified	Number of identifications of each species made by direct MALDI-TOF from a single blood culture bottle using:					
	Cut off value ≥ 1.700			Cut off value ≥ 2.000		
	Saponin	SDS	Sepsi-Typer	Saponin	SDS	Sepsi-Typer
Gram positives						
*Actinomyces oris*	1	1	1	0	0	0
*Clostridium innocuum*	1	0	0	0	0	0
*Corynebacterium amycolatum*	1	1	1	0	1	0
*Corynebacterium mucifaciens*	0	1	0	0	0	0
*Corynebacterium tuberculostearicum*	0	0	1	0	0	0
*Enterobacter cloacae*	3	3	3	2	3	3
*Enterococcus faecalis*	4	4	4	0	2	3
*Enterococcus faecium*	2	7	7	1	7	7
*Granulicatella adiacens*	0	1	1	0	1	1
*Kocuria rhizophila*	0	1	1	0	0	0
*Micrococcus luteus*	2	3	3	1	2	2
*Propionibacterium acnes*	0	2	3	0	1	2
*Staphylococcus aureus*	18	17	17	12	16	17
*Staphylococcus capitis*	13	11	13	8	7	6
*Staphylococcus cohnii*	0	1	1	0	1	1
*Staphylococcus epidermidis*	23	19	17	11	8	7
*Staphylococcus haemolyticus*	2	2	2	0	1	2
*Staphylococcus hominis*	11	10	11	8	10	10
*Streptococcus agalactiae*	1	1	1	0	1	1
*Streptococcus anginosus*	1	0	0	0	0	0
*Streptococcus constellatus*	1	1	2	0	0	0
*Streptococcus dysgalactiae*	0	1	1	0	0	0
*Streptococcus intermedius*	0	1	1	0	0	0
*Streptococcus lutetiensis*	0	1	1	0	0	0
*Streptococcus mutans*	0	2	1	0	1	1
*Streptococcus parasanguinis*	0	1	1	0	0	1
*Streptococcus pneumoniae*	0	1	1	0	1	1
*Streptococcus pyogenes*	0	3	3	0	2	3
*Streptococcus sanguinis*	1	4	4	0	2	3
Gram negatives						
*Bacteroides fragilis*	2	0	0	2	0	0
*Escherichia coli*	32	35	36	28	34	36
*Klebsiella oxytoca*	3	3	3	3	3	3
*Klebsiella pneumoniae*	5	5	5	4	5	5
*Moraxella catarrhalis*	1	1	1	0	1	1
*Morganella morganii*	2	2	2	2	2	2
*Proteus mirabilis*	4	4	4	3	3	4
*Pseudomonas aeruginosa*	3	2	3	3	2	2
*Serratia marcescens*	3	2	3	1	2	3
*Serratia ureilytica*	0	1	0	0	1	0
No identification	59	44	40	110	79	72
Total	199	199	199	199	199	199

Cut off value ≥1.700 = ≥1.700 and top three results matching

Of the 181 positive cultures, five failed to grow on sub-culture. Of the 15 direct MALDI-TOF results from these bottles, 14 gave no identification. In one bottle, *Corynebacterium mucifaciens* was identified with a score of 1.729 using SDS. Gram positive bacilli had been seen in the Gram stain.

Thirty-two of the 181 positive blood cultures showed polymicrobial growth (≥2 different species) on subculture. Two or more organisms were seen on the Gram stain in only 3/32 (9%) bottles with at least one organism seen in all 32 bottles. In 12/32 (38%) samples both organisms grown would have had the same morphology on Gram stain. Where direct MALDI-TOF identifications were obtained for polymicrobial culture bottles, MALDI-TOF only ever identified one organism from a bottle. Using Saponin, MALDI-TOF identified a single organism in 11/32 (34%) polymicrobial bottles using ≥2.000 and 20/32 (63%) bottles using ≥1.700 and the top three matches were concordant as a cut-off score respectively. Using SDS, a single organism was identified in 21/32 (66%) or 24/32 (75%) bottles respectively. Using SepsiTyper, a single organism was identified in 22/32 (69%) or 24/32 (75%) bottles respectively. Where obtained, the direct MALDI-TOF identifications were always concordant with the single organisms identified in the Gram stain and at least one of the organisms seen in Gram stains with two organisms seen.

One hundred and forty-four positive blood cultures from 100 patients had one organism identified from conventional subculture (monomicrobial). These were selected for further detailed analysis to identify the yield of identifications with direct MALDI-TOF and the concordance of these direct identifications with conventional identifications from sub-cultured organisms. Direct MALDI-TOF identifications using both cut off strategies for monomicrobial bottles were compared with organism identifications from subcultures. Each direct MALDI-TOF result was thus determined to be concordant with subculture, discordant with subculture or “No ID” where no reliable identification using the cut off was obtained. These results, broken down by bacterial groups and cut off score are shown in Table 2.

**Table 2:** Direct MALDI-TOF results in monomicrobial cultures

Bacterial Group	Cut off value	Identified using Saponin (%)			Identified using SDS (%)			Identified using SepsiTyper (%)			Total
		Concordant	Discordant	No ID	Concordant	Discordant	No ID	Concordant	Discordant	No ID	
Staphylococci and Micrococci	≥2.000	33 (50)	0 (0)	33 (50)	35 (53)	0 (0)	31 (47)	37 (56)	0 (0)	29 (44)	66
	≥1.700	55 (83)	1 (2)	10 (15)	51 (77)	1 (2)	14 (21)	51 (77)	1 (2)	14 (21)	66
Enterobacteriaceae	≥2.000	27 (77)	0 (0)	8 (23)	32 (91)	0 (0)	3 (9)	34 (97)	0 (0)	1 (3)	35
	≥1.700	30 (86)	0 (0)	5 (14)	33 (94)	0 (0)	2 (6)	33 (94)	0 (0)	2 (6)	35
Streptococci and Enterococci	≥2.000	0 (0)	0 (0)	18 (100)	9 (50)	0 (0)	9 (50)	10 (56)	0 (0)	8 (44)	18
	≥1.700	4 (22)	0 (0)	14 (78)	9 (50)	0 (0)	9 (50)	10 (56)	0 (0)	8 (44)	18
Other Gram negatives	≥2.000	9 (69)	0 (0)	4 (31)	8 (62)	0 (0)	5 (38)	8 (62)	0 (0)	5 (38)	13
	≥1.700	10 (77)	0 (0)	3 (23)	8 (62)	0 (0)	5 (38)	9 (69)	0 (0)	4 (31)	13
Other Gram positives	≥2.000	0 (0)	0 (0)	12 (100)	2 (17)	0 (0)	10 (83)	2 (17)	0 (0)	10 (83)	12
	≥1.700	1 (8)	0 (0)	11 (92)	2 (17)	0 (0)	10 (82)	3 (25)	1 (8)	8 (67)	12
Total	≥2.000	69 (48)	0 (0)	75 (52)	86 (60)	0 (0)	58 (40)	91 (63)	0 (0)	53 (37)	144
	≥1.700	100 (69)	1 (1)	43 (30)	103 (72)	1 (1)	40 (28)	106 (74)	2 (1)	36 (25)	144

Concordance and discordance are with identification by conventional culture and identification

Cut off value ≥1.700 = ≥1.700 and top three results matching

In the 144 monomicrobial cultures, using ≥2.000 as the cut-off value, the lowest yield of identification to species level was obtained with Saponin and the highest yield was obtained using SepsiTyper (Table 2). No discordances between the organisms identified by direct MALDI-TOF and the corresponding report from routine diagnostic culture were observed using ≥2.000 as the cut-off value.

The proportion of samples with identification to species level was increased by amending the MALDI-TOF cut-off value to ≥1.700 and top three results matching. However, two discordances from two samples were observed (Table 2). These discordances were further investigated; in the first sample *Corynebacterium tuberculostearicum* was identified by MALDI-TOF using the SepsiTyper method only (also confirmed using PCR and sequencing of the 16S gene from the blood culture) but the culture results had been reported as “*Propionibacterium* sp.”. Further examination of laboratory notes indicated that the identification of a *Propionibacterium* sp. was made on colony and Gram morphology only indicating that the reported culture result may have been imprecise and that there was no true discordance. In the second discrepant sample the three MALDI-TOF preparation methods and 16S PCR with sequencing all consistently detected *Staphylococcus epidermidis* in the aerobic bottle and *Actinomyces oris* in the anaerobic bottle but the culture results had been reported as “*S. epidermidis*”. Further examination of laboratory notes indicated that a faint growth of another organism had been noted on anaerobic plates but identification was not pursued as the isolate was not thought to be clinically significant, again indicating that the reported culture result was incomplete and that there was no true discordance.

The difference in yield of culture concordant identifications by direct MALDI-TOF using both cut-off strategies was evaluated with the McNemar test using a significance level of P < 0.05. None of the differences in yield between methods using ≥1.700 and top three results matching were statistically significantly different in this evaluation (Saponin vs. SDS P = 0.8445; Saponin vs. SepsiTyper P = 0.4414; SDS vs. SepsiTyper P = 0.5465). Using the ≥2.000 scoring system, the difference in yield between SDS and SepsiTyper was not statistically significant (P = 0.2278). Differences between Saponin versus SDS or Saponin versus SepsiTyper were significant (P = 0.0125 and P = 0.0015 respectively).

## Discussion

This study is the first to perform a three way comparison of the SepsiTyper kit with two detergent lysis methods for preparing positive blood cultures for direct MALDI-TOF analysis prospectively in a clinical setting.

Using a cut off score of ≥2.000 (i.e. the manufacturer’s recommended cut off value for identification of organisms from culture plates) there was no statistical difference found between the rates of obtaining sub-culture concordant species level identifications using 10% SDS or SepsiTyper to prepare monomicrobial positive blood cultures for direct MALDI-TOF. Rates of identification were 60% and 63% respectively. The use of 5% Saponin with a cut off score of ≥2.000 was found to give significantly fewer reliable species level MALDI-TOF identifications from direct blood cultures that either of the other methodologies.

Using a cut off score of ≥1.700 and top three results matching, there was no statistical difference found between the rates of obtaining sub-culture concordant species level identifications using 10% SDS, 5% saponin or SepsiTyper to prepare monomicrobial positive blood cultures for direct MALDI-TOF. The rate of identification for all three methods ranged from 69 to 74% using this lower cut off. With all three lysis methods, the lower score cut off value of ≥1.700 and top three results matching increased the proportion of species level identifications by at least 12% more when compared to the more stringent ≥2.000 cut off value but did however yield two apparent discordances between MALDI-TOF and conventional culture. Both apparent discordances were subsequently found to be due to incomplete identification of cultured organisms and the results obtained by MALDI-TOF were correct and corroborated by 16S PCR and sequencing.

We have demonstrated that up to 72% of bacteria in monomicrobial blood cultures can be identified on day one by MALDI-TOF. However polymicrobial cultures remain problematic for MALDI-TOF directly from blood culture as previously reported [11, 17]. In this study, where a direct identification was obtained from a culture that was polymicrobial on sub-culture, the identification was always consistent with one of the cultured organisms. This suggests that the direct identifications from polymicrobial cultures, when obtained with the methods described, can be considered accurate for the organism identified (despite not giving identification of all organisms) when making empirical clinical decisions, whilst awaiting technical confirmation. In common with several other studies [5, 6] we found a higher proportion of identification by MALDI-TOF to species level for blood cultures containing Gram negative bacteria than Gram positive bacteria and reduced rates of successful identification for streptococci.

The performance of the SepsiTyper kit has been evaluated in at least 21 reported studies as recently reviewed by Morgenthaler and Kostrzewa [6]. The reported percentage identification to species level in monomicrobial cultures is variable across the studies from 56 to 100% and probably reflects the different methodologies and gold standards with which it was compared as well as possible differences in the proportions of different bacteria isolated from bacteraemic patients at different sites. When compared with alternative methods, SepsiTyper has been found to be superior to gel separator tubes [18], differential centrifugation [5, 13, 19] and another commercial preparation kit [19]. In agreement with this study, SepsisTyper has previously been found to comparable with methods using lysis with saponin [4, 20] and SDS [5]. There are no previous reports comparing both Saponin and SDS.

For a clinical diagnostic laboratory, it is attractive to select the convenience of a commercially available, validated kit. However, the performance of any methodology will always need to be locally verified and operating costs are an increasingly important consideration. At the time of this evaluation, the cost per sample of the ready-made SDS or saponin reagent was 100 times lower than the cost of the Sepsityper kit with identical consumption of other consumables and staff time.

Identification of bacteria in the clinical diagnostic laboratory by MALDI-TOF is still a developing technology. As MALDI-TOF is used increasingly as a direct identification method from blood cultures the databases and algorithms that support the identifications are likely to improve. During this study, the investigators interpreted the MALDI-TOF results in isolation from the Gram results and any clinical information. If this method were implemented, the results would be interpreted in the light of laboratory expertise, the Gram stain and clinical information which have been shown to improve correct identifications and minimise misidentifications [5, 7, 21, 22]. Results from this study also suggest that the cut off score value to be selected for clinical use needs to balance the benefits of a greater number of identifications on day one against the risks of possible misidentification, especially in the case of polymicrobial cultures. A number of studies have demonstrated clinical benefits from earlier direct identification of organisms in blood cultures by using MALDI-TOF [17, 23, 24]. One of our aims for the development of direct MALDI-TOF on positive cultures within our laboratory is to assess the potential clinical impact on patient care.

## Conclusions

This study has demonstrated that two relatively inexpensive in-house detergent lysis methods are non-inferior when compared with a commercially available kit for preparing positive blood cultures for direct MALDI-TOF in a large clinical diagnostic microbiology laboratory. Using a MALDI-TOF score cut off value of ≥1.700 and top three results matching for species level identification gave reliable organism identifications from 72% of monomicrobial blood cultures and identified one of the organisms from polymicrobial cultures in 34–75% of samples depending on the method. No organisms were identified by MALDI-TOF in any samples that were truly discordant with the culture results.

## Abbreviations

MALDI-TOF: matrix-assisted laser desorption/ionization time-of-flight mass spectrometry
SDS: sodium dodecyl sulfate
HCCA: α-cyano-4-hydroxycinnamic acid
PHE: Public Health England
